# Nonclinical cardiovascular safety of pitolisant: comparing International Conference on Harmonization S7B and Comprehensive *in vitro* Pro‐arrhythmia Assay initiative studies

**DOI:** 10.1111/bph.14047

**Published:** 2017-10-19

**Authors:** Xavier Ligneau, Rashmi R Shah, Isabelle Berrebi‐Bertrand, Gary R Mirams, Philippe Robert, Laurent Landais, Pierre Maison‐Blanche, Jean‐François Faivre, Jeanne‐Marie Lecomte, Jean‐Charles Schwartz

**Affiliations:** ^1^ Bioprojet‐Biotech Saint‐Grégoire France; ^2^ Pharmaceutical Consultant Gerrards Cross UK; ^3^ Centre for Mathematical Medicine and Biology, School of Mathematical Sciences University of Nottingham Nottingham UK; ^4^ Hôpital Bichat Paris France; ^5^ Laboratoire Signalisation et Transports Ioniques Membranaires Université de Poitiers‐CNRS Poitiers France

## Abstract

**Background and Purpose:**

We evaluated the concordance of results from two sets of nonclinical cardiovascular safety studies on pitolisant.

**Experimental Approach:**

Nonclinical studies envisaged both in the International Conference on Harmonization (ICH) S7B guideline and Comprehensive *in vitro* Pro‐arrhythmia Assay (CiPA) initiative were undertaken. The CiPA initiative included *in vitro* ion channels, stem cell‐derived human ventricular myocytes, and *in silico* modelling to simulate human ventricular electrophysiology. ICH S7B‐recommended assays included *in vitro* hERG (K_V_11.1) channels, *in vivo* dog studies with follow‐up investigations in rabbit Purkinje fibres and the *in vivo* Carlsson rabbit pro‐arrhythmia model.

**Key Results:**

Both sets of nonclinical data consistently excluded pitolisant from having clinically relevant QT‐liability or pro‐arrhythmic potential. CiPA studies revealed pitolisant to have modest calcium channel blocking and late I_Na_ reducing activities at high concentrations, which resulted in pitolisant reducing dofetilide‐induced early after‐depolarizations (EADs) in the ICH S7B studies. Studies in stem cell‐derived human cardiomyocytes with dofetilide or E‐4031 given alone and in combination with pitolisant confirmed these properties. In silico modelling confirmed that the ion channel effects measured are consistent with results from both the stem cell‐derived cardiomyocytes and rabbit Purkinje fibres and categorized pitolisant as a drug with low torsadogenic potential. Results from the two sets of nonclinical studies correlated well with those from two clinical QT studies.

**Conclusions and Implications:**

Our findings support the CiPA initiative but suggest that sponsors should consider investigating drug effects on EADs and the use of pro‐arrhythmia models when the results from CiPA studies are ambiguous.

**Abbreviations**
APA: amplitude of the action potential
APD: action potential duration
CI: confidence interval
CiPA: Comprehensive *in vitro* Proarrhythmia Assay
EADs: early after‐depolarizations
hERG: human ether‐a‐go‐go potassium channel
ICH: International Conference on Harmonization
iPSC: induced pluripotent stem cell
QTc: QT interval corrected for heart rate
QTcF: QTc using the Fridericia formula
RMP: resting membrane potential
TdP: torsade de pointes
TQT: thorough QT
UTA: U‐wave to T‐wave amplitude
Vmax: maximal velocity of depolarization

## Introduction

In 2005, the International Conference on Harmonization (ICH) adopted two guidelines, requiring all new drugs to be evaluated for their effects on the QT interval of the surface ECG. The nonclinical guideline ICH S7B (ICH, 2005a) recommends an *in vitro* study on the **K_v_11.1 channel** (hERG), responsible for the major outward repolarizing current (I_Kr_), and an *in vivo* study to be followed, if necessary, by other investigations such as a repolarization assay measuring action potential parameters in a suitable *in vitro* preparation and the use of pro‐arrhythmia models. The other guideline, ICH E14 (ICH, 2005b), recommends a clinical thorough QT (TQT) study. However, both guidelines focus on a drug's effect on QT interval, rather than its pro‐arrhythmic risk. These guidelines are thought to be inadequate, because they disregard the important role of ion channels other than K_v_11.1 (hERG) channels in modulating pro‐arrhythmogenic potential. The TQT study is cost‐ineffective (Bouvy *et al.,* 2012) and aims to quantify precisely an effect (drug‐induced increase in QTc interval) that correlates poorly with the actual risk of pro‐arrhythmia (Haverkamp *et al.,* 2001; Shah and Morganroth, 2015). A positive nonclinical and/or clinical QT‐signal often leads to termination of the drug from further development (Stockbridge *et al.,* 2013).

Consequently, a new paradigm, referred to as the ‘Comprehensive *in vitro* Proarrhythmia Assay’ (CiPA), is under consideration for more efficient characterization of a drug's pro‐arrhythmic potential (Sager *et al.,* 2014). This paradigm recognizes the critical role of multiple ion channels blockade and calls for two sets of studies consisting of (a) an *in vitro* study of a drug's effects on multiple ion channels, followed by incorporating these effects in an *in silico* model of human ventricular electrophysiology to predict a drug's pro‐arrhythmic potential and (b) confirmation of these predictions *in vitro* using human induced pluripotent stem cell (iPSC)‐derived cardiomyocytes (Mirams *et al.,* 2014; Sager *et al.,* 2014; Gintant *et al.,* 2016). CiPA also calls for intensive ECG monitoring in at least one phase I clinical study (that includes an active control) which, coupled with exposure‐response analysis, could provide information comparable to that from a TQT study (Shah and Morganroth, 2012; Darpo and Garnett, 2013). The CiPA package is expected to be presented to the ICH E14/S7B discussion group by December 2017 (Colatsky *et al.,* 2016).

Therefore, we undertook a range of ICH S7B‐ and CiPA‐compliant studies to carefully characterize the cardiac safety profile of pitolisant and to provide data useful for further progressing the CiPA initiative. Pitolisant (also known as BF2.649) is a novel, highly potent and selective histamine H_3_ receptor antagonist/inverse agonist, with a *K*_i_ value between 0.3 and 2.4 nM at the human receptor, which enhances histaminergic transmission in the brain and thereby promotes wakefulness (Ligneau *et al.,* 2007). In narcolepsy, pitolisant significantly enhances wakefulness, thereby reducing sleepiness and cataplexy episodes in the orexin^−/−^ mouse narcolepsy model as well as in humans (Lin *et al.,* 2008; Dauvilliers *et al.,* 2013; Szakacs *et al.,* 2017). With 96% protein‐binding (in house data), the maximal mean total pitolisant serum concentrations are in the order of ~100 ng·mL^−1^ (i.e. 0.30 μM, corresponding to a free drug concentration of 0.012 μM) at steady state after a 2 week treatment with 40 mg pitolisant hydrochloride maximal therapeutic dose. Pitolisant, designated as an orphan drug, was granted a marketing authorization by the European Medicines Agency in March 2016 for the treatment of narcolepsy with or without cataplexy. In the present manuscript, we summarize the results of more recent CiPA‐style nonclinical studies with pitolisant and compare these with the findings from earlier ICH S7B nonclinical studies.

## Methods

### Compliance with requirements for studies using animals

#### Validity

Cardiovascular *in vivo* investigations were performed according to ICH S7A and ICH S7B guidance recommendations.

#### Ethics, animal protection and statistics

Animal studies are reported in compliance with the ARRIVE guidelines (Kilkenny *et al*., 2010; McGrath and Lilley, 2015). All animals were used in compliance with the French legislation regarding the protection of laboratory animals based successively on EU Directives 1986/609/EU and 2010/63/EU and approved by Ethical Committees C2EA‐79 (Bioprojet‐Biotech) and C2EA‐60 (Contract Research Organization), when applicable. The numbers of animals used were determined taking into account the known variability of the parameters measured and as few as possible to obtain the necessary information.

#### Housing and husbandry

SPF New Zealand rabbits (8–13 weeks old, 2.1–2.7 kg; Centre Lago, 01540 Vonnas, France) were housed separately in standard conditions [20 ± 2°C, humidity 50 ± 20%, 20 changes of air (by volume) an hour with filtered, non‐recycled air, 12/12 h light/dark cycle, light on at 07:00 h] in polycarbonate housing units with perforated floor (0.51 m^2^, height 45 cm, no bedding) with food (Code 110, Safe, 89120 Augy, France) and 0.22 μm filtered water *ad libitum*. Conventional Beagle dogs (12–20 months old, males 10–15 kg, nulliparous females 10–13 kg, CEDS, 89120 Mézilles, France) were housed in single sex groups (three dogs per kennel, 16.2 m^2^, no bedding) with free access to food (Code 125, Safe, 89290 Augy, France) and water. Animals were allowed to play in groups in a large pen once a day. Dogs were checked for comfort and health every day. After surgery, and during measurements as appropriate, dogs were housed in individual cages (floor area: 1.20 m^2^, height: 1.8 m, i.e. regulatory cage size according to the 86/609/CEE directive in force at the time of the study) under artificial lighting (12 h) between 07:00 and 19:00 h in a controlled ambient target temperature of 18 ± 3°C. During periods between testing sessions, dogs were kept in groups in the kennel.

### *In vitro* study on K_v_11.1 (hERG) channel current: acute and long‐term effects

This study, already required under ICH S7B, investigated the effects of pitolisant (0.1, 0.3, 1, 3, 10 and 100 μM) on K_v_11.1 tail current in HEK‐293 cells stably transfected with hERG cDNA and maintained in an appropriate culture medium. A whole‐cell patch clamp study was performed on isolated cells at room temperature. Cells were initially clamped at −80 mV, and biphasic pulses (4.8 s at +20 mV and then for 5 s at −50 mV) were applied at a 0.067 Hz frequency. After initial stabilization of the preparation, vehicle for control, increasing concentrations of pitolisant and 100 nM of E‐4031 (the reference positive compound) were perfused until equilibrium, which was reached in approximately 10 min for each condition in three to four cells per condition. Peak tail current amplitudes for each concentration were measured and averaged for four consecutive stimuli. Vehicle control tested in parallel evaluated the spontaneous run‐down of hERG current over time in testing conditions and the corresponding correction was applied.

Although the hERG channel assay is considered to be the method of choice in safety pharmacology for investigating QT‐related risk assessment, it may fail to identify substances which have an indirect effect (and thus prolong QT interval) through inhibition of hERG protein trafficking from the endoplasmic reticulum to the cell membrane, thus highlighting a possible limitation of acute short‐term screening.

Consequently, the effect of pitolisant on hERG channel trafficking was investigated *in vitro* by studying effects on peak tail current amplitude after long‐term (over 24 h) application of pitolisant to HEK‐293 cells stably transfected with hERG cDNA. The concentration used (1 μM) was close to its hERG IC_50_ value reported above. The effect of pitolisant was compared to that of pentamidine (10 μM), a reference compound known to reduce this current by inhibiting hERG trafficking, after a long exposure and without any acute effect. The test after chronic pitolisant exposure, with no noticeable degradation of the drug substance, was performed without any pitolisant (i.e. replacement of the cell culture medium by a fresh pitolisant‐free medium just before the patch clamp test). In parallel, acute effects of pitolisant (0.3 and 1 μM) and pentamidine (10 μM) were also measured. Whole‐cell patch clamp conditions were similar to the previous one with the following slight changes (biphasic pulses 2 s at +20 mV and then for 3.2 s at −50 mV applied at a 0.1 Hz).

### *In vitro* studies on other ion channels

The whole‐cell patch clamp technique was used to investigate the effect of pitolisant on major cardiac currents, namely peak and late I_Na_ (Na_V_1.5), I_to_ (K_V_4.3), I_Ks_ (K_V_7.1/mink), I_K1_ (K_ir_2.1), I_Kur_ (K_V_1.5), I_CaL_ (Ca_V_1.2) and I_CaT_ (Ca_V_3.2), currents under consideration as part of the CiPA initiative. HEK‐293 or CHO cells were stably transfected with cDNA of the different ion channels and maintained in appropriate culture medium. As shown in Table 1, the effect of pitolisant on each ion current was studied in four cells per ion channel by whole‐cell patch clamp in standard testing conditions (see Supporting Information Tables S1 and S2) using standard drugs as the positive controls (with an assay‐to‐assay variability within ±5% in their inhibitory responses for the various ion channel assays).

**Table 1:** Effects of pitolisant on human cardiac ion channel currents

Ion channel	Channel current	Pitolisant IC_50_ (μM)	Fitted IC_50_ (μM)	Reference compound and % inhibition
Outward repolarizing currents				
K_V_11.1	I_Kr_ (hERG)	1.3	1.3	E‐4031 (100 nM): 86%
K_V_7.1/mink	I_Ks_	>10	No effect	Mefloquine (10 μM): 61%
K_V_1.5	I_Kur_	≥10	13.8	Terfenadine (10 μM): 81%
K_V_4.3	I_to_	~10	11.4	Dapoxetine (30 μM): 74%
Inward depolarizing currents				
Na_V_1.5 resting state	I_Na_	>10	26.4^a^	Lidocaine (10 mM): 99%
Na_V_1.5 fast inactivated state	I_Na_	≥10	26.4^a^	Lidocaine (10 mM): 99%
Na_V_1.5 slow inactivated state	I_Na_	≥10	26.4^a^	Lidocaine (10 mM): 93%
Ca_V_1.2	I_CaL_	~10	9.5	Nifedipine (1 μM): 88%
Ca_V_3.2	I_CaT_	≥10	25.7	Mibefradil (1 μM): 83%
K_ir_2.1	I_K1_	≥10	16.5	ML 133 (10 μM): 96%

The ‘Fitted IC_50_’ column gives the values which were used as inputs into the in silico action potential models, after fitting Hill curves, with Hill coefficients of one, through the available data points.

a Fits to fast and slow inactivated Na_v_1.5 yielded 26.3 and 26.5 μM, respectively, and so the mean 26.4 μM was used for the whole Na_v_1.5 current.

### *In silico* action potential modelling

Mathematical models of cardiac electrophysiology that can provide predictions of the combined effect of multiple ion channel blockade (Mirams *et al.,* 2011) have been proposed as a tool for cardiac safety testing (Mirams *et al.,* 2012; Sager *et al.,* 2014), and the reader is referred to these references for an introduction to these models. The IC_50_ values, fitted to ion channel data and summarized in Table 1, formed the input into our models for simulating action potentials in cardiomyocytes. We ran simulations of ion channel block caused by pitolisant using the ‘ApPredict’ Web Portal (Williams and Mirams, 2015), with predictions of torsadogenic risk using the model proposed by Grandi *et al*. (2010) and a method proposed earlier (Mirams *et al.,* 2011) to predict torsadogenic risk according to categories recommended by Redfern *et al*. (2003). We used the Shannon *et al*. (2004) model for comparison with rabbit‐based experiments. We used these models rather than the O'Hara‐Rudy model (O'Hara *et al.,* 2011) suggested by CiPA (Colatsky *et al.,* 2016) as we had previously validated Grandi model‐based predictions of torsade risk in response to multiple‐ion channel block (Mirams *et al.,* 2011), and the Shannon *et al*. (2004) model is rabbit‐specific. In the future, both Grandi and O'Hara models may require some ‘rebalancing’ of currents to improve predictions of human drug‐induced responses (Mann *et al.,* 2016).

### *In vitro* effects in rabbit Purkinje fibres

Following a lethal dose of pentobarbital, the hearts of male New Zealand rabbits were immediately excised and immersed in a modified Tyrode solution maintained at room temperature and previously saturated with O_2_/CO_2_ (95%/5%). A piece of ventricle containing free running Purkinje fibres was fixed to the silastic bottom of an organ bath perfused with the same solution maintained at 37°C for 30 min. The preparation was then superfused with the modified Tyrode solution and stimulated for 30 min at 2 Hz and then at 1 Hz (2 ms stimulus at twice threshold). Action potentials were acquired and analysed to provide measures of various electrophysiological parameters such as the resting membrane potential (RMP), maximal velocity of depolarization (Vmax), amplitude of the action potential (APA) and action potential duration measured at 50 and 90% of repolarization (APD_50_ and APD_90_). After a stabilization period of at least 1 h, action potentials typical for Purkinje fibres were recorded. Action potential reproducibility and stability were confirmed during a further 20 min stabilization period. The preparation was then superfused with experimental solutions which included 0.1, 1 and 10 μM pitolisant and 0.1 μM **dofetilide** alone or together with 10 μM pitolisant. Each was applied for 20 min to enable steady state to be reached. In addition, the emergence of early after‐depolarizations (EADs), favoured by calcium loading during the late phase 2 of the prolonged action potential, is recognized as a better marker of pro‐arrhythmic potential than APD prolongation alone. Hence, we investigated the effects of 1, 3 and 10 μM of pitolisant in rabbit Purkinje fibres that exhibited induced EADs and triggered activity [manifested *in vivo* as torsade de pointes (TdP) and/or ventricular tachycardia] caused by 0.1 μM dofetilide, a prototype class III antiarrhythmic drug.

### *In vitro* studies in human stem cell‐derived cardiomyocytes

Action potentials in isolated human cardiomyocytes were recorded with pitolisant, E‐4031 or dofetilide and their combination using a conventional patch‐clamp technique in whole‐cell configuration at room temperature (22 ± 2°C). Human cardiomyocytes (Cor.4U®, Axiogenesis AG, Cologne, Germany) were derived from human iPSC. Preparations were stimulated at 0.5 Hz with 4 ms rectangular pulses from 1 to 1.5 times depolarization threshold to −60 mV. We studied RMP, APA, action potential durations measured at 20 and 90% repolarization (APD_20_ and APD_90_) and APD_90_‐APD_20_. Experiments were carried out on 19 cells originating from 19 different culture dishes:

- Four cells for each of three concentrations (1, 3 or 10 μM) of pitolisant,
- three cells for 0.1 μM E‐4031 followed by pitolisant at 10 μM and
- four cells for 0.1 μM dofetilide followed by pitolisant at 10 μM.

### In *vivo* studies

Three *in vivo* studies using p.o. and/or i.v. doses of pitolisant were carried out in rabbits and dogs to characterize further their *in vivo* electrocardiographic effects and pro‐arrhythmic potential.

#### Effect of i.v. infusion in anaesthetized rabbits

Since substantial clinical evidence indicates a greater susceptibility of females to developing TdP, the study preferentially included female animals with 22 (81%) of 27 total animals studied being females. Male animals were allocated to the reference‐treated group (one out of four animals, 75% female rabbits) and to the 1 mg·kg^−1^ single‐dose pitolisant‐treated group (four out of seven animals, 43% female rabbits). All the other experimental groups were 100% female. Animals were anaesthetized with sodium pentobarbital (45–60 mg·kg^−1^ i.v.), maintained on a heated pad and mechanically ventilated with room air. Before surgery and during the experiment, depth of anaesthesia was confirmed by demonstrating lack of jaw tone and palpebral and corneal reflexes. A fluid‐filled catheter was inserted into the carotid artery to measure systemic arterial blood pressure and for blood sampling. A venous catheter was implanted into the jugular vein for infusion of drugs. Standard surface ECG leads I, II and III were monitored continuously to measure ECG intervals and the occurrence of ventricular tachyarrhythmia. Measurements of arterial blood pressures were calculated over 30 s duration at baseline and every 5 min. For automated ECG capture and measurements, specific libraries of ECG complexes were built per animal constituted of 13 ECG complexes measured manually at 5 min intervals. Then, for all determinations, the means of 10–30 consecutive sinus beats were automatically calculated at each time point (i.e. at the end of the stabilization period and then every 5 until 60 min after starting drug infusion) or until the occurrence of premature ventricular beats. Parameters recorded were systolic, diastolic and mean arterial blood pressure (mmHg), heart rate (beats·min^‐1^ calculated from RR interval) as well as PR, QRS and QT/QTU intervals (ms), amplitudes of T‐wave and U‐wave (mV) and rhythm disturbances retrieved from ECG signals. The QT interval was corrected for heart rate using the formula developed for rabbits (Carlsson *et al.,* 1993) and adapted to in‐house conditions [corrected QT = QT‐0.666∙(RR‐221) in ms]. The ratio of U‐wave to T‐wave amplitude (UTA ratio), an ECG index of pro‐arrhythmic potential, was calculated to normalize the individual variations in repolarization according to Gbadebo *et al*. (2002). Ventricular arrhythmia was defined according to Lambeth conventions (Walker *et al.,* 1988) with TdP defined as >7 consecutive beats of polymorphic ventricular tachycardia. Arrhythmia were scored according to the following scale: 0 being no arrhythmia, 1 being premature beats, 2 being auriculo‐ventricular blocks, 3 being ventricular tachycardia and 4 being TdP; and scores correspond to the average of maximal life‐threatening arrhythmias recorded during the experiment. After a 10 min stabilization period, the rabbits received i.v. over 60 min either pitolisant as a slow infusion (1 mg·kg^−1^ single dose or up to 10 mg·kg^−1^ dose range, i.e. 1, 3.3 and 10 mg·kg^−1^ administered over 20 min successive steps) or clofilium, a selective I_Kr_ blocker as a positive reference, up to 1 mg·kg^−1^ dose range. In a second set of experiments, rabbits received either pitolisant (3 mg·kg^−1^ single dose) or its vehicle (3.3 mL·kg^−1^·h^−1^ sterile water) over 30 min and were monitored post‐infusion over a further 30 min washout period. The range of doses was appropriate for exploring the effects of a wide range of drug exposures, including maximal concentrations far above those expected at therapeutic doses. Haemodynamic and ECG recordings were monitored continuously over 60 min following the start of infusion.

#### Effect in anaesthetized methoxamine‐sensitized (Carlsson) rabbit model

The anaesthetized **methoxamine**‐sensitized rabbit model of TdP is frequently used to study torsadogenic potential of drugs (Carlsson *et al.,* 1990). Briefly, female animals were pre‐sensitized with i.v. infusion of methoxamine, an **α_1_‐adrenoceptor** agonist, delivered at 15 μg·kg^−1^·min^−1^ (6 mL·h^−1^) over 70 min. Ten minutes later, pitolisant or clofilium was administered as a slow i.v. infusion, at the rate of 50 or 150 μg·kg^−1^·min^−1^ over 1 h (i.e. 3 or 9 mg·kg^−1^ full dose over 1 h) or 20.5 μg·kg^−1^·min^−1^ (i.e. 1.2 mg·kg^−1^ full dose over 1 h), respectively. In addition, another set of experiments investigated effects of the combination of pitolisant (50 μg·kg^−1^·min^−1^) with clofilium (20.5 μg·kg^−1^·min^−1^). The selected range of doses ensured that we explored effects of a wide range of drug exposures, including maximal concentrations far above those expected at therapeutic doses.

#### Effect of single p.o. and i.v. doses in conscious telemetered dogs

Six Beagle dogs (three males and three females) were used to evaluate the cardiovascular effects of pitolisant in freely moving animals. Telemetry implantation was performed as previously described (Lacroix *et al.,* 2003). Approximately 2 weeks later, blood pressure, heart rate and ECG intervals were recorded per blocks of 30 s at the time points of interest for 30 min before and for 24 h after administration of test compound. ECG traces were inspected visually for any rhythm or conduction abnormalities (such as arrhythmia, tachyarrhythmia, atrioventricular conduction blocks), and values of PR, QRS and QT intervals were obtained by a validated manual reading procedure (mean calculated from three non‐consecutive sinus beats). The QT interval measured was corrected for heart rate using the Fridericia formula (QTcF) and the van de Waters formula. Cardiovascular effects of pitolisant were investigated in two sessions. Five to six dogs (two or three males and three females) were studied with at least 48 h between each test session.

The first session tested pitolisant given p.o. at 5, 10 and 15 mg·kg^−1^ compared to the vehicle (2 mL·kg^−1^ of water) with measurement of pitolisant serum levels 1 and 4 h after dosing. At the highest dose of 15 mg·kg^−1^ p.o., this route provided an exposure (59 ± 25 ng·mL^−1^), without any adverse CNS events (i.e. pre‐convulsive signs) in this species, close to the exposure recorded in humans at therapeutic doses. Therefore, the second session tested pitolisant given i.v. as a single bolus at 1.5 and 4.5 mg·kg^−1^ (maximal dose by this route was without any adverse CNS events) in comparison to the vehicle (sterile saline at 0.2 mL·kg^−1^) with pitolisant serum levels measured 0.5 and 2 h after dosing but also in a full pharmacokinetic independent session (2, 5, 15 and 30 min and 1, 2, 4, 8 and 24 h post‐dose) to allow a higher drug exposure. Pitolisant serum levels were assessed by LC‐mass spectrometry using a validated analytical method.

### Data and statistical analyses

The data and statistical analyses comply with the recommendations on experimental design and analysis in pharmacology (Curtis *et al.,* 2015). Statistical analyses were performed with Statview version 5.0 (SAS, Cary, NC, USA), GB Stat version 6.5 (Dynamic Microsystems, Silver Spring, MD, USA) or SigmaStat 3.0 (Systat, San Jose, CA, USA), and in all analyses, *P* < 0.05 was taken as indicative of statistical significance. Figure legends provide the group size (*n*) and specific statistical tests performed when *n* was at least five per group. For all data, *n* corresponds to independent values. For ethical reasons (3R rules), the number of animals was reduced to three to four individuals in some experiments because the effects were clear enough. In the study with cardiomyocytes, the magnitude of the effects observed on APDs (together with corresponding small error bars) did not warrant increasing the group size above four. No randomization was performed because of the small group sizes. All parameters stated are the numerical values measured, which were not influenced by any observer‐related bias and, therefore, blinding was not considered to be necessary. For clarity, most values presented in the figures are normalized to control/baseline values to minimize unwanted sources of variation. Statistical analyses were performed on absolute values measured and not on percentage changes.

### Nomenclature of targets and ligands

Key protein targets and ligands in this article are hyperlinked to corresponding entries in http://www.guidetopharmacology.org, the common portal for data from the IUPHAR/BPS Guide to PHARMACOLOGY (Southan *et al.,* 2016), and are permanently archived in the Concise Guide to PHARMACOLOGY 2015/16 (Alexander *et al.,* 2015a,b).

## Results

### *In vitro* study on K_v_11.1 (hERG) channel current

Pitolisant inhibited hERG channel current with an IC_50_ value of 1.3 μM, thus indicating that it lacks any significant hERG channel blocking activity at concentrations of 0.3 μM or less, and the reference compound (E‐4031) performed as expected with an IC_50_ value of 10.7 nM (Table 1). After long‐term exposure to pitolisant (1 μM) over a 24 h period and following a washout of the incubation medium, there was no significant effect on hERG current measured without pitolisant in the test medium, and a ratio of peak tail current amplitude over cell capacitance of 62 ± 4 versus 55 ± 5 pA∙pF^−1^ in control. There were no significant changes in pitolisant concentrations in the cell culture medium over the 24 h of exposure as evidenced by LC‐mass spectrometry. As expected, pentamidine (10 μM), a reference compound known to inhibit hERG trafficking, significantly inhibited the hERG tail current amplitude by 57%, a value in good agreement with the literature (Kuryshev *et al.,* 2005). Thus, chronic exposure to pitolisant, at least at concentrations up to 1 μM, lacked any effect on hERG channel trafficking from the endoplasmic reticulum to the cell membrane.

### *In vitro* studies on other ion channels

Pitolisant was found to have IC_50_ values of around or above 10 μM for inhibition of Na_V_1.5 (I_Na_), K_V_4.3 (I_to_), K_V_7.1/mink (I_Ks_), K_ir_2.1 (I_K1_), K_V_1.5 (I_Kur_), Ca_V_1.2 (I_CaL_) and Ca_V_3.2 (I_CaT_) channels. IC_50_ values of 26.4 μM for Na_V_1.5 and 9.5 μM on Ca_V_1.2 were fitted compared with 1.3 μM for K_V_11.1 (I_Kr_) (Table 1).

### *In silico* action potential modelling

**Figure 1:**
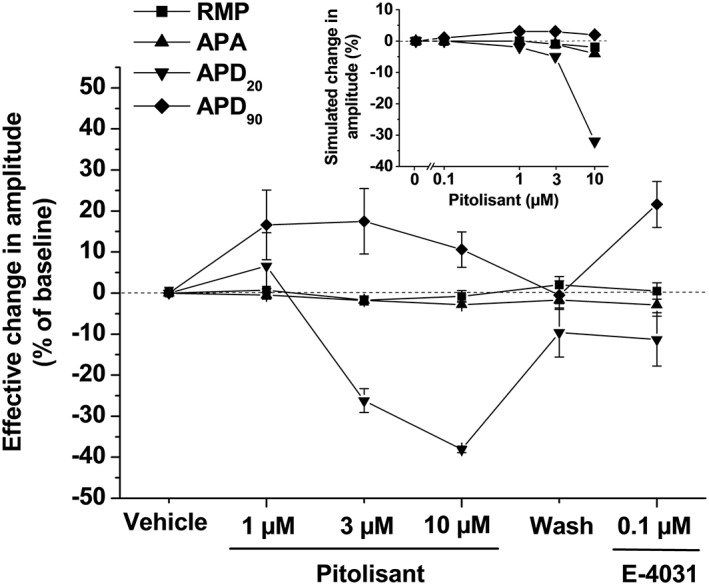
Effects of E‐4031 and pitolisant on action potential parameters from human stem cell‐derived ventricular cardiomyocytes compared to simulated effects of pitolisant on human cardiomyocyte action potential parameters using the Grandi *et al*. model at 1 Hz (inset). Changes represent mean ± SEM of *n* percentages calculated from individual variation of each parameter for each cell versus vehicle values (*n* = 4 cells for vehicle and pitolisant, *n* = 2 for E‐4031). Parameters are RMP, APA and action potential durations measured at 20 and 90% of repolarization (APD_20_ and APD_90_) with corresponding absolute values for the vehicle at baseline being 86 ± 2 mV, 130 ± 2 mV, 330 ± 21 ms and 604 ± 61 ms respectively.

The simulated effects on action potential parameters for human at 1 Hz can be seen in the inset of Figure 1. Very little effect of pitolisant is seen on RMP and APA, as observed in the experimental stem cell‐derived cardiomyocyte assay. There was little effect on the APDs at concentrations up to 1 μM (well above clinically relevant total drug concentrations, i.e. 0.24 μM at the highest therapeutic dose) and very similar patterns compared to experimental APD_20_ and APD_90_. This suggests that the ion channel screening panel we studied has captured the major effects of the compound as these predictions are in agreement with experimental results observed in stem cell‐derived cardiomyocytes. This modelling result provides additional confidence in predicting the overall cardiac safety of pitolisant in the clinic. The model was used to predict a ‘Redfern Risk Category’ of pro‐arrhythmic risk (Redfern *et al.,* 2003) for pitolisant by a method described previously (Mirams *et al.,* 2011). Our results categorize pitolisant in Redfern Category 4 (low risk) at clinical concentrations. We also predicted the effect of pitolisant in rabbit ventricular cells at 1 Hz to compare with those recorded in rabbit Purkinje fibre. The simulation results, shown in Supporting Information Figure S1, are in broad agreement with the experimental results, described below, confirming that the known ion channel effects are consistent with all the available data.

### *In vitro* effects in rabbit Purkinje fibres

**Figure 2:**
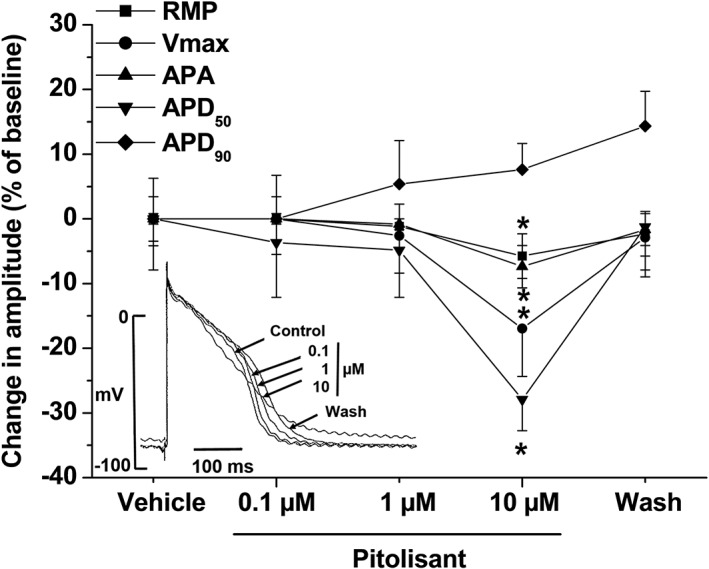
Changes induced by pitolisant in action potential parameters in rabbit Purkinje fibre. Action potential parameters were measured after 20 min of equilibration for each concentration of pitolisant. Basal values of RMP, Vmax, APA and action potential durations measured at 50% and 90% of repolarization (APD_50_ and APD_90_, respectively) were −87 ± 3 mV, 727 ± 30 V·s^−1^, 122 ± 1 mV, 165 ± 13 ms and 223 ± 14 ms respectively. Values are expressed as mean ± SEM changes (as % baseline values) of *n* = 6 experiments. Statistics (ANOVA followed by multiple comparison using the Sidak procedure).

At a concentration of 1 μM, pitolisant had only minimal effects on electrophysiological parameters of the action potential in rabbit Purkinje fibres (Figure 2), confirming the results obtained with the *in vitro* studies on other ion channels (see section above). At a higher concentration of 10 μM, there was a non‐significant increase (+9 ± 5% vs. the control) in APD_90_ and a significant decrease in APD_50_ (−27 ± 4% vs. the control). In addition, there were significant decreases in resting RMP (−6 ± 1% vs. the control), in APA (−8 ± 3%) and in Vmax (−17 ± 5%), suggesting that at high concentrations, pitolisant may have an effect on sodium current generally, not only late but also peak I_Na_. These effects were reversible following washout except for APD_90_ prolongation. These findings suggest that at high concentrations above free therapeutic plasma levels, pitolisant exerts an inhibitory effect on I_Kr_ during phase 3 of the action potential (as evidenced by an increase in APD_90_) and on L‐type calcium channels (as evidenced by the decrease of 27% in APD_50_, corresponding to an estimated IC_50_ value of about 5 μM for APD_50_).

#### Effect on early after‐depolarizations and triggered activity

**Figure 3:**
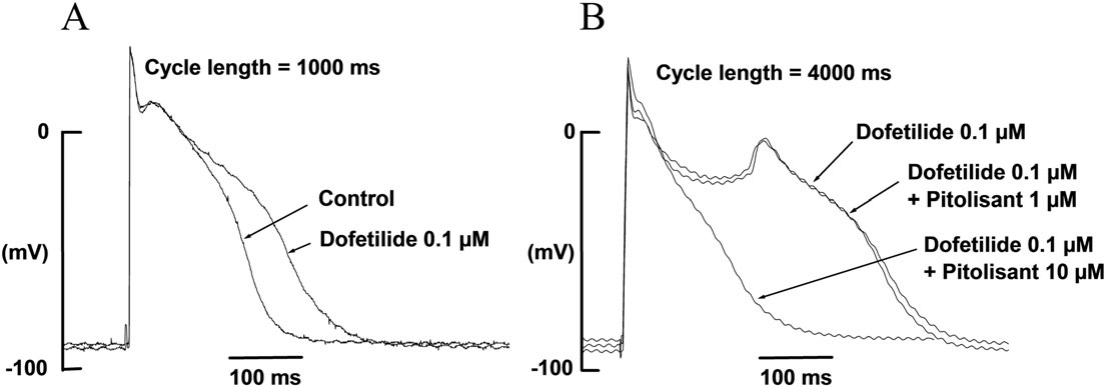
Effect of pitolisant on dofetilide‐induced early after depolarization (EAD) in rabbit Purkinje fibre. (A) Traces were recorded before and 20 min after dofetilide application (0.1 μM) at a stimulation rate of 1 Hz. (B) Traces were recorded at a stimulation rate of 0.25 Hz. Action potential recorded in the presence of dofetilide 0.1 μM exhibited a pronounced early after depolarization (application of dofetilide 0.1 μM for 35 min; APD_90_ = 593 ms). Addition of 1 μM pitolisant did not suppress the EAD (applications of dofetilide 0.1 μM for 55 min and pitolisant 1 μM for 20 min; APD_90_ = 598 ms). In contrast, the EAD disappeared in the presence of pitolisant 10 μM (applications of dofetilide 0.1 μM for 1 h10 and pitolisant 10 μM for 15 min; APD_90_ = 275 ms).

Dofetilide consistently and predictably induced EADs or triggered activity in all 10 preparations, and pitolisant had a dose‐related EAD‐mitigating effect (Figure 3). Application of 1 μM of pitolisant had no effect (in four out of four experiments) while 3 μM was effective in inhibiting dofetilide‐induced EADs in two out of three experiments. Pitolisant might be suspected to favour the occurrence of EADs because of triangulation of action potential shape (see Figures 2 and 3). However, 10 μM of pitolisant always suppressed dofetilide‐induced EADs in all seven experiments and dofetilide‐induced spontaneous triggered activity in one out of one experiment. Pitolisant eliminated, rather than exacerbated, the pro‐arrhythmic effect of dofetilide.

### In vitro studies in human stem cell‐derived cardiomyocytes

**Figure 4:**
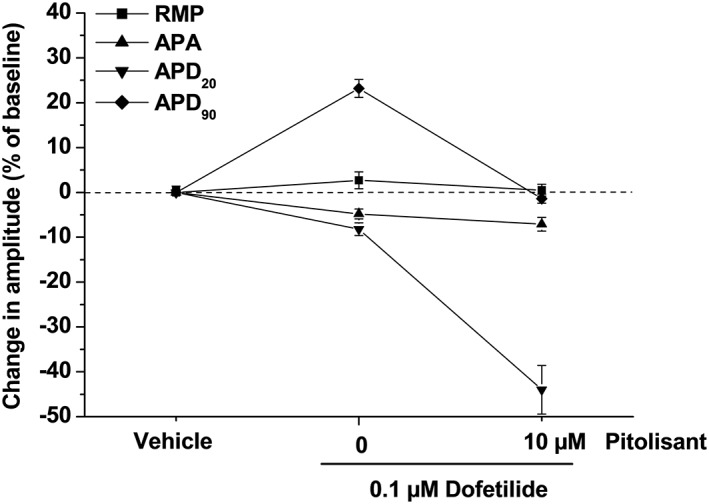
Effects of dofetilide alone or in combination with pitolisant on action potential parameters from human stem cell‐derived ventricular cardiomyocytes. Changes represent mean ± SEM of *n* percentages calculated from individual variation of each parameter for each cell versus vehicle values (*n* = 4 cells for vehicle, dofetilide alone and combination of dofetilide and pitolisant). Parameters are RMP, APA and action potential durations measured at 20 and 90% of repolarization (APD_20_ and APD_90_) with corresponding absolute values for the vehicle at baseline being 88 ± 3 mV, 125 ± 4 mV, 344 ± 54 ms and 635 ± 75 ms respectively.

Results from this study showed that pitolisant had little effect on RMP and APA. Results for APD_20_ and APD_90_ (Figures 1 and 4) and APD_90_‐APD_20_ (not shown) showed that

- Pitolisant had a concentration‐dependent effect of decreasing APD_20_, suggesting L‐type calcium channel block at high concentrations (≥3 μM).
- Pitolisant prolonged APD_90_, suggesting some hERG channel inhibition at concentrations ≥1 μM. At higher concentrations (10 μM), the effect on APD_90_ was much attenuated, suggesting compensatory effects of pitolisant mediated by action(s) on additional ion channel(s).
- Pitolisant at 3 μM elicited a maximal increase of 97 ms in APD_90_ compared to the effect (increase of 120 ms) of 0.1 μM of E‐4031, a classical potent hERG channel blocker. Effect of higher concentrations of pitolisant on APD_90–20_ (increase of 192 and 176 ms vs. baseline at 3 and 10 μM compared to the increase of 253 ms for 0.1 μM of E‐4031) was consistent with concentration‐dependent effects on hERG and L‐type calcium channels.
- Effects of dofetilide on APD_20_ and APD_90_ were consistent with the data described for this compound (mainly an increase in APD_90_).
- At a high concentration, pitolisant potentiated the small effect of dofetilide on APD_20_. Interestingly, pitolisant also fully reversed dofetilide‐induced increase in APD_90_, suggesting perhaps a calcium channel blocking activity.

### *In vivo* studies

As described in the Methods, three *in vivo* studies in two different species (rabbit and dog), using p.o. and/or i.v. doses of pitolisant, were carried out to further characterize their *in vivo* electrocardiographic effects, including any pro‐arrhythmic signals. Regarding drug exposure, it is worth emphasizing that the protein binding of pitolisant is about 96% in all animal species studied and in humans (in‐house data).

#### Effect of i.v. infusion in anaesthetized rabbits

Effects of pitolisant (up to 10 mg·kg^−1^ by slow i.v. infusion) were compared to those of clofilium, a selective I_Kr_ blocker (up to 1 mg·kg^−1^). Pitolisant, when given as a single dose (1 mg·kg^−1^ over 60 min), did not cause any significant change in heart rate, QTc interval (+3 ± 1%) or blood pressure (data not shown). After cumulative infusion with 1, 3.3 and 10 mg·kg^−1^ in successive 20 min steps (i.e. 50, 160 and 500 μg·kg^−1^·min^−1^), ensuring a maximal total drug exposure of 4140 ± 450 ng·mL^−1^ (i.e. total drug concentration of 13.8 ± 1.5 μM which corresponds to free drug concentration of ~0.55 μM), heart rate decreased by 17 ± 4%. However, no prolongation of QTc interval was recorded (2 ± 1% vs. baseline, Table 2). In contrast, clofilium induced a prolongation of QTc interval (+13 ± 5% vs. baseline, Table 2) and other ECG abnormalities (shortening of PR interval and prolongation of QRS interval – data not shown). Pitolisant elicited some changes in the terminal portion of repolarization (appearance of U waves) in some ECGs. However, although the incidence of U waves was higher, there was no major change in the UTA ratio (data not shown). Ventricular arrhythmias were never observed in the vehicle and in pitolisant‐treated (up to 10 mg·kg^−1^ by i.v. slow infusion) groups evidencing a low pro‐arrhythmic potential of pitolisant. In contrast, clofilium infusion induced severe ventricular tachyarrhythmia; none of these animals remained in sinus rhythm at the end of the experiment. The ventricular tachycardia incidence was 100% (*n* = 4/4 experiments), and TdP occurred in 75% of experiments (*n* = 3/4) in the clofilium‐treated group. Interestingly, pitolisant (10 mg·kg^−1^) abolished ventricular premature beats, ventricular tachycardia and TdP induced by clofilium (1 mg·kg^−1^) in this preparation (data not shown).

**Table 2:** Effects of infusions of pitolisant or clofilium on QTc interval (ms) in anaesthetized rabbits

Dose (*n*)		Clofilium	Pitolisant	
		1 mg·kg^−1^	1 mg·kg^−1^	10 mg·kg^−1^ dose range
		(4)	(7)	(4)
		QTc interval (ms) (mean ± SEM)		
Baseline		152 ± 2	152 ± 3	144 ± 4
Time (min) into infusion	10	152 ± 2	154 ± 2	148 ± 3
	20	157 ± 3	156 ± 2	149 ± 2
	30	168 ± 5	156 ± 2	151 ± 2
	40	172 ± 8	156 ± 2	151 ± 1
	50	–	157 ± 2	148 ± 4
	60	–	157 ± 3	147 ± 2

Clofilium was infused over 1 h at a dose of 1 mg·kg^−1^. Pitolisant was infused over 1 h at the dose of 1 mg·kg^−1^ or successively over 20 min at 1, 3.3 and 10 mg·kg^−1^. QT interval was corrected for variation in heart rate using the formula developed for rabbits (Carlsson *et al*., 1993) and adapted to in‐house conditions [QTc = QT‐0.666·(RR‐221) with QT and RR durations in ms], QTc interval values provided are mean ± SEM.

#### Effect in anaesthetized methoxamine‐sensitized (Carlsson) rabbit model

**Figure 5:**
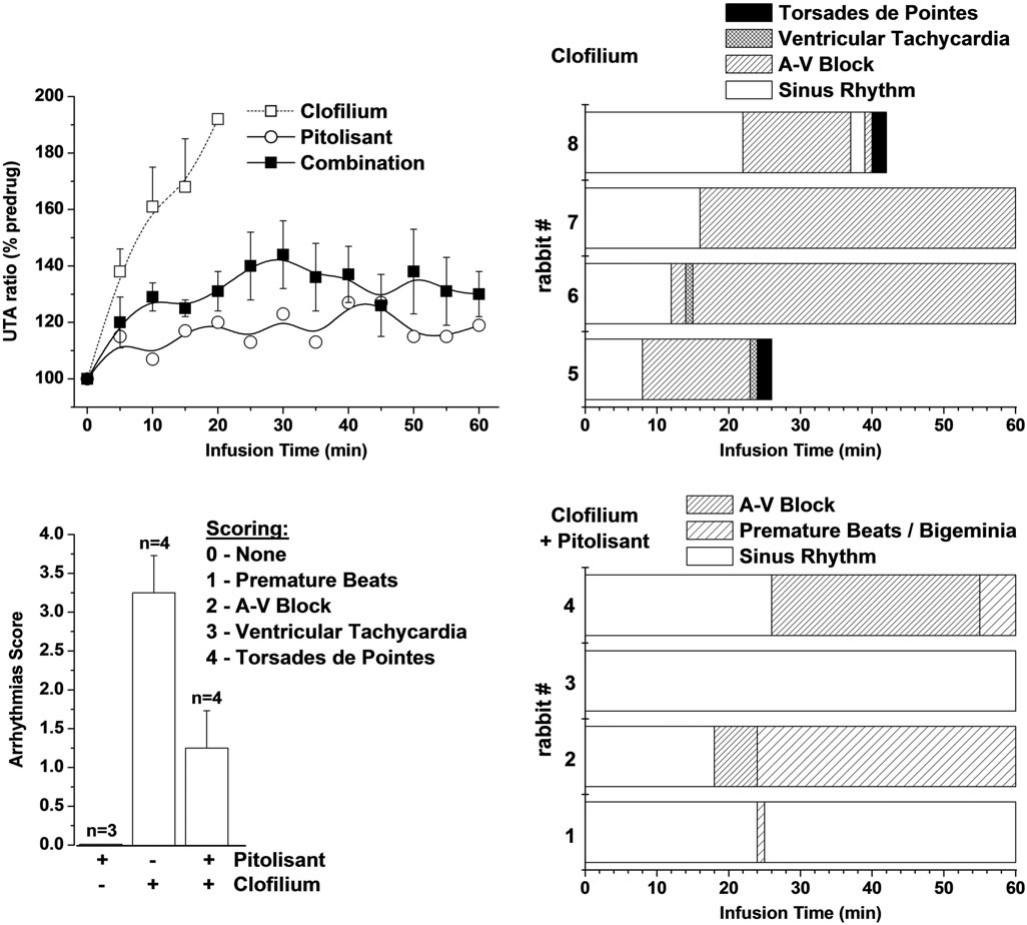
Incidence of ventricular arrhythmias in methoxamine‐sensitized rabbits infused with clofilium tosylate, pitolisant and their combination. Ventricular arrhythmias were assessed according to the Lambeth conventions in groups treated with pitolisant (50 μg·kg^−1^·min^−1^), clofilium tosylate (20.5 μg·kg^−1^·min^−1^) or the combination of both drugs. (Left) Mean ± SEM UTA ratio (% of pre‐drug value) over time (top) and arrhythmia score evaluated for each experimental group (bottom). (Right) Horizontal bars are schematic representation of occurrence of rhythm abnormalities over time for each clofilium‐treated rabbit (#1 to #8), in the absence (top) or in the presence (bottom) of concomitant pitolisant infusion.

**Figure 6:**
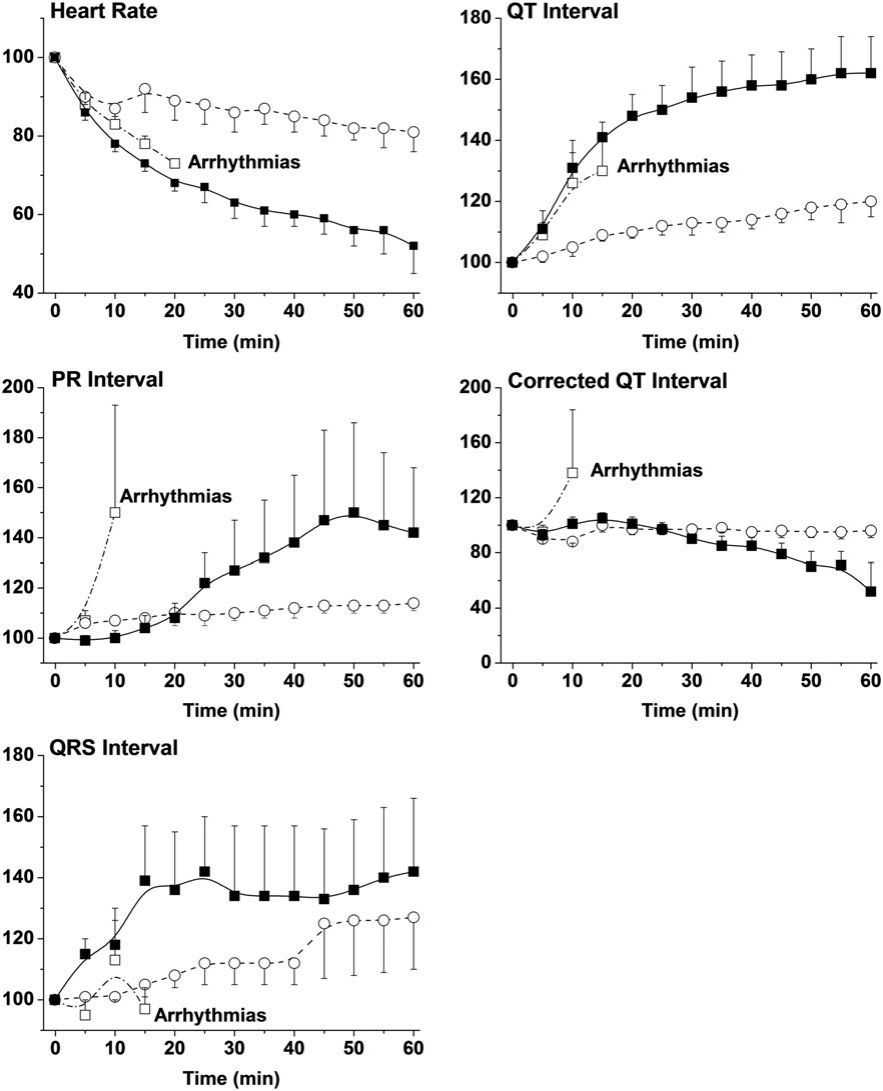
Effects of clofilium tosylate, pitolisant and their combination on heart rate and ECG parameters in methoxamine‐sensitized anaesthetized rabbits. Mean ± SEM changes in ECG parameters (when measurable) during the 60 min infusion period in methoxamine‐treated rabbits. QT interval was corrected for variation in heart rate using the formula developed for rabbits (Carlsson *et al*., 1993) and adapted to in house conditions [QTc = QT‐0.666·(RR‐221) with QT and RR durations in ms]. Open circles: pitolisant 50 μg·kg^−1^·min^−1^(*n* = 3 animals); open squares: clofilium tosylate 20.5 μg·kg^−1^·min^−1^(*n* = 4); solid squares: a combination of both (*n* = 4).

In this model, pitolisant alone was devoid of any effect on QTc interval, and it lacked any pro‐arrhythmic effect even at high total drug concentrations (1062 ± 125 and 3387 ± 1226 ng·mL^−1^, i.e. 3.5 ± 0.4 and 11.3 ± 4.1 μM, corresponding to free drug concentrations of ~0.14 and 0.45 μM, after the 50 and 150 μg·kg^−1^·min^−1^ 1 h infusion, respectively) well above the concentrations achieved clinically. There were, however, minor morphological changes as demonstrated by a small increase in UTA ratio (Figure 5). In contrast, clofilium rapidly induced ECG changes (bradycardia, prolongation of QTc interval and a marked 90% increase in UTA ratio) that resulted in ventricular tachyarrhythmia (Figures 5 and 6). Administration of a combination of pitolisant and clofilium elicited no change in QTc interval but a marked bradycardia (Figure 6). Interestingly, administration of pitolisant concurrently with clofilium normalized the UTA ratio, which was almost doubled under clofilium alone. Furthermore, the pro‐arrhythmic potential of clofilium was reduced by combining it with pitolisant (Figure 5).

#### Effect of single p.o. and i.v. doses in conscious telemetered dogs

**Figure 7:**
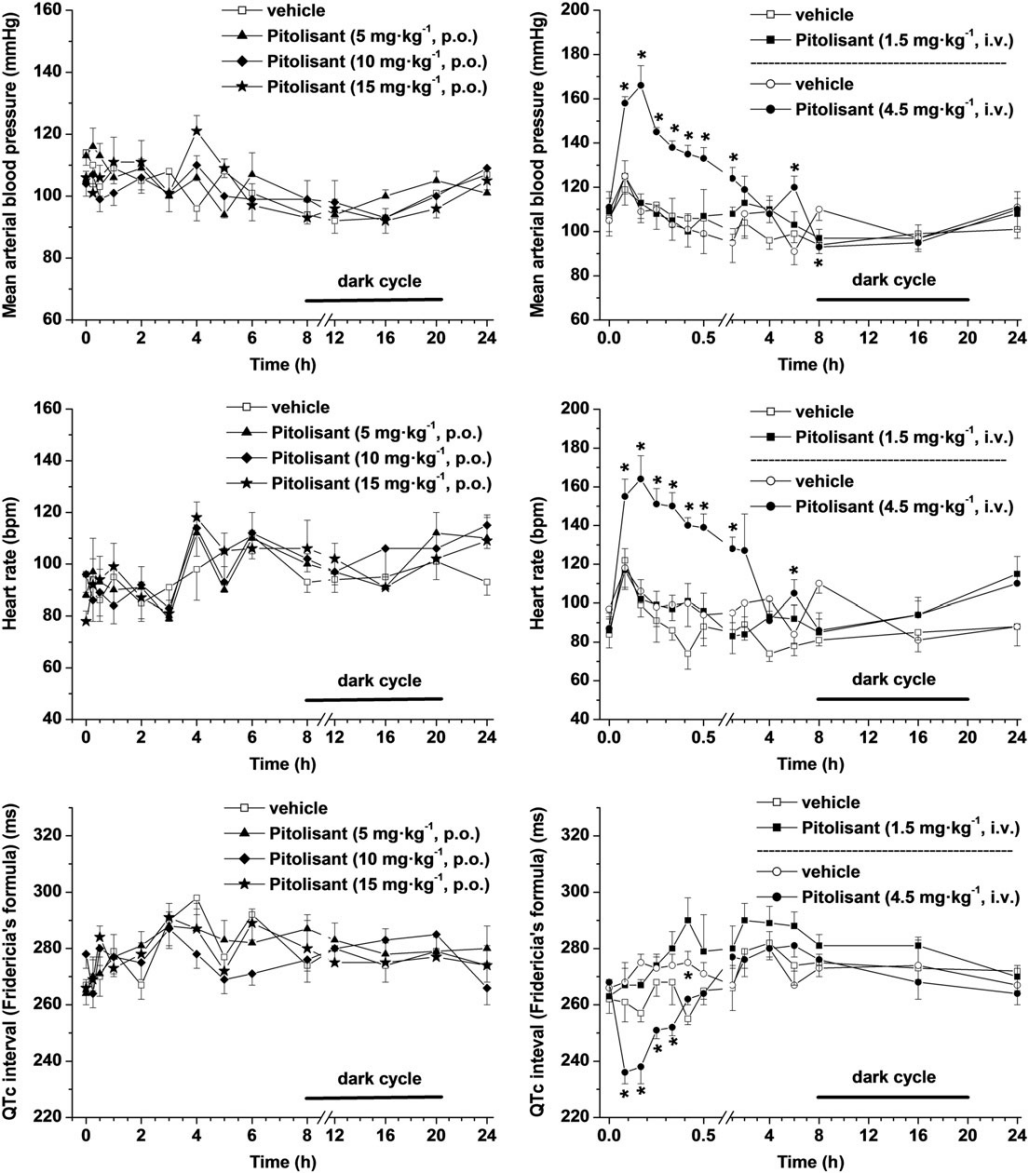
Haemodynamic and repolarization effects following p.o. and i.v. doses of pitolisant in conscious telemetry‐implanted dogs. Changes in mean arterial blood pressure, heart rate and QTcF interval following p.o. (panels on left) and i.v. (panels on right) doses of pitolisant and the vehicle. Mean ± SEM of values recorded in *n* = 6 dogs. Statistics (ANOVA followed by a Dunnett's test).

Compared to the vehicle, single doses of pitolisant (5, 10 and 15 mg·kg^−1^ p.o. or 1.5 mg·kg^−1^ i.v.) had no effect on heart rate, mean arterial blood pressure, QTc interval (Figure 7) or any other ECG parameter (data not shown). Blood samples collected 1 and 4 h after dosing demonstrated relatively low total drug exposure of animals receiving pitolisant p.o. (3.5 ± 1.0, 15.8 ± 5.8 and 59.2 ± 24.6 ng·mL^−1^ at 1 h after the administration of 5, 10 and 15 mg·kg^−1^ pitolisant, respectively) and a high variability between dogs. The exposure in animals receiving the i.v. dose of 1.5 mg·kg^−1^ was satisfactory, with total drug concentrations reaching 294 ± 40 ng·mL^−1^ at 5 min and 231 ± 36 ng·mL^−1^ at 30 min.

Much higher exposure was achieved after the i.v. dose of 4.5 mg·kg^−1^ with total drug concentrations reaching 1044 ± 570 ng·mL^−1^ (3.48 ± 1.90 μM) at 5 min, 579 ± 187 ng·mL^−1^ (1.93 ± 0.62 μM) at 30 min and 159 ± 49 ng·mL^−1^ (0.53 ± 0.16 μM) at 4 h (bearing in mind that total drug concentration of 3.5 μM corresponds to free drug concentration of ~0.14 μM). This resulted in increases in mean arterial blood pressure and heart rate for up to 4 h after injection (Figure 7). It produced a decrease in QTcF interval for 20 min with a return to baseline 30 min after injection (Figure 7). Thereafter, there was no significant difference between the vehicle and pitolisant for a period up to 24 h. Use of van de Water's formula to correct the measured QT interval resulted in findings (not shown) similar to those following the application of Fridericia correction. No arrhythmia or other morphological changes in the ECG attributable to pitolisant were observed over the test period in 6/6 dogs. Exposure to high total drug concentration of pitolisant could account for the hypertension and tachycardia observed at the 4.5 mg·kg^−1^ i.v. dose as pitolisant had some activity at α_2_‐adrenoceptors with *K*_i_ values of 2.9, 4.3 and 1.2 μM for **α_2A_**, **α_2B_** and **α_2C_‐adrenoceptors** respectively. The transient decrease in QTcF interval, although statistically significant, is likely spurious, resulting from marked increase in heart rate since, in presence of large changes in heart rate, there is much less confidence in the Fridericia corrected QTc intervals.

## Discussion and conclusions

Nonclinical results on pitolisant across both ICH S7B and CiPA initiative studies are consistent and, as discussed hereafter, correlate well with data from clinical trials.

The ICH S7B studies showed that pitolisant has a minimal hERG channel blocking activity at concentrations up to 0.3 μM. Its IC_50_ value for hERG blockade is 1.3 μM which corresponds to a safety margin around 100, assuming 96% protein‐binding (in house data) and maximal mean total pitolisant serum concentrations of ~100 ng·mL^−1^ (i.e. 0.30 μM, corresponding to a free drug concentration of 0.012 μM) at steady state after a 2 week treatment with the 40 mg pitolisant hydrochloride maximal therapeutic dose (Lin *et al.,* 2008). This safety margin is typical for QT prolonging drugs which lack a pro‐arrhythmic potential. In addition, no effect on the hERG current was measured in absence of pitolisant following pre‐test exposure to pitolisant for 24 h showing that pitolisant does not inhibit hERG channel trafficking. Recently, Obejero‐Paz *et al*. (2015) have suggested that an hERG trafficking assessment should be added to the CiPA package. *In vitro* ion channel studies, performed according to CiPA‐style proposals, suggested pitolisant to have modest calcium channel blocking as well as late I_Na_ reducing activities. These ion channel data, integrated using an *in silico* electrophysiology model, elicited an action potential for pitolisant rather similar to the one experimentally recorded in human ventricular cardiomyocytes. This, together with the high IC_50_ values (> or ~10 μM) of pitolisant for these channels relative to the hERG channel (IC_50_ ~ 1.3 μM), suggests that high concentrations of pitolisant are required for a clinically relevant block of these channels to mitigate the pro‐arrhythmic effect of any hERG blockade. In this context, supra‐therapeutic concentrations of pitolisant prevented dofetilide‐induced increase in APD_90_ in human iPSC cardiomyocytes. Our cardiomyocyte results are in line with data from larger assessments of iPSC cardiomyocytes for evaluating drug‐induced arrhythmias proposed as part of the CiPA initiative, that is, APD prolongation and arrhythmia with hERG channel blocking drugs and APD shortening and prevention of arrhythmia with calcium channel blocking drugs (Blinova *et al.,* 2017).

Both calcium channel blocking as well as late I_Na_ reducing activities have already been reported to reduce any risk of proarrhythmia due to hERG channel blockade and QT interval prolongation. Hence, to mitigate the pro‐arrhythmic effect of hERG blockade, the IC_50_ values for the L‐type calcium and/or the late I_Na_ currents should be fairly close to the one for I_Kr_. Examples are ranolazine (Antzelevitch *et al.,* 2014; Crumb *et al.,* 2016), verapamil (Obejero‐Paz *et al.,* 2015; Crumb *et al.,* 2016) and mexiletine (Gao *et al.,* 2013; Antzelevitch *et al.,* 2014; Gualdani *et al.,* 2015; Crumb *et al.,* 2016). There are no well‐documented reports of TdP in association with their use.

In contrast, the development of 400 mg vanoxerine in the treatment of atrial fibrillation had to be discontinued because of unacceptably high prevalence of TdP (Piccini *et al.,* 2016). Its IC_50_ value for hERG channel was just over ninefold greater than that for the late I_Na_ current (9.3 and 85.2 nM, respectively; Obejero‐Paz *et al.,* 2015). We note that vanoxerine is 140‐fold more potent than pitolisant in inhibiting the hERG channel, and clinically, it induced dose‐dependent increases in QTcF interval (mean of 32.2 ms on 200 mg dose, 51.3 on 300 mg and 60.7 ms on 400 mg) (Dittrich *et al.,* 2015).

Importantly, however, there is insufficient information regarding the degree of block of the L‐type calcium and reduction in late I_Na_ currents required *in vivo* for a clinically meaningful and beneficial effect in terms of reducing pro‐arrhythmic risk. Recent *in vitro* studies have shown that mexiletine inhibits late I_Na_ with an IC_50_ value of 17.6 ± 1.9 μM and fast I_Na_ with an IC_50_ value of 34.6 ± 2.9 μM (Gao *et al.,* 2013) and hERG channel with an IC_50_ value of 3.7 ± 0.7 μM (Gualdani *et al.,* 2015). In a study in canine Purkinje fibres, erythromycin induced a pronounced prolongation of APD and the appearance of EADs in all Purkinje preparations (9/9). After addition of mexiletine (10 mM), erythromycin‐induced prolongation of APD shortened markedly and EADs disappeared (7/9) (Fazekas *et al.,* 1998). The therapeutic concentrations of mexiletine range from 3.4 to 9.5 μM, and yet, it is effective in type 3 congenital LQTS (Mazzanti *et al.,* 2016). Badri *et al*. (2015) have shown mexiletine to be also effective in acquired LQT syndrome. In this context, we note that Johannesen *et al*. (2016) have reported that their ion channel patch clamp experiments suggest that both mexiletine and lidocaine induce only about 20% block of late sodium current at the concentrations that caused approximately 20 ms shortening of QTc. Even ranolazine, at clinical C_max_, induces only 21% block, compared to only 3% block by quinidine, of late I_Na_ (Vicente *et al.,* 2015). Equally, at clinical concentrations, pitolisant does not have any meaningful effect on hERG channels that requires mitigation. However, the possibility that pitolisant may have a clinically relevant effect on L‐type calcium channel or late I_Na_ at concentrations lower than those suggested by its IC_50_ values cannot be ruled out. IC_50_ values determined in *in vitro* systems are variable (Elkins *et al.,* 2013) and often do not correlate with *in vivo* activity (Makielski, 2016). Plasma concentrations far lower than IC_50_ concentrations are known to induce QT interval prolongation. In one simulation study, only a 10% hERG blockade by dofetilide corresponded to a QT prolongation of 20 ms [95% confidence interval (CI): 12–32 ms] (Jonker *et al.,* 2005). Similarly, *in vivo* in humans, smaller effects on calcium and late I_Na_ at lower concentrations (IC_15–20_ in the range of 0.5–1 μM) may be effective in clinically relevant reduction of the currents concerned. As noted earlier, application of 1 μM of pitolisant had no effect in inhibiting dofetilide‐induced EADs, but 3 μM was partially effective.

These results were in line with those obtained previously in rabbit Purkinje fibres. In this ICH S7B‐compliant follow‐up model, pitolisant exerted inhibitory effect on I_Kr_ current and on L‐type calcium channel at high concentrations as evidenced by the increase in the APD_90_ and the decrease in the APD_50_, respectively and eliminated EADs induced by dofetilide despite its apparent propensity to provoke triangulation of action potential shape.

*In vivo* nonclinical studies in anaesthetized rabbits and telemetered dogs revealed either no or inconsistent effects on the QTc interval. No arrhythmias were observed in any *in vivo* animal studies. The study in Carlsson's rabbit model further confirmed the lack of a pro‐arrhythmic effect, where pitolisant did not prolong QTc interval or induce any life‐threatening arrhythmia; indeed, it actually reduced the incidence of clofilium‐induced proarrhythmia. In line with inhibitory potencies of pitolisant at the various cardiac ion channels, data from these studies also support the conclusion that pitolisant has no effect on PR and QRS intervals, and little or no QT‐prolonging activity, at therapeutic mean maximal total drug concentrations of ~100 ng·mL^−1^ (i.e. 0.30 μM, corresponding to a 0.012 μM free concentration) at steady state after a 2 week treatment with 40 mg pitolisant hydrochloride maximal therapeutic dose. At higher doses which result in concentrations up to 55 times higher than these, pitolisant has ancillary electrophysiological activity that can antagonize the pro‐arrhythmic effect of other drugs.

The results from both sets of nonclinical studies (ICH S7B‐compliant and CiPA‐style) enabled categorization of pitolisant as a drug with low risk torsadogenic potential (the principal objective of the CiPA initiative). Indeed, the CiPA‐style studies provided tentative evidence for pitolisant to have inhibitory effects on calcium and/or late I_Na_ currents which could account for its lack of pro‐arrhythmic effects, even if the inhibition of these currents, compared to the inhibition of hERG current, is relatively small. The CiPA findings were consistent with data from earlier extensive nonclinical follow‐up studies conducted as recommended in ICH S7B, both confirming its lack of pro‐arrhythmic potential.

Results from both sets of nonclinical studies are also in good agreement with the effect of pitolisant on QT interval observed in the clinical SAD and TQT studies (Shah *et al.,* 2016). Analysis by ICH E14‐recommended intersection union test revealed comparable maximum mean (90% CI) placebo‐corrected increase in QTcF interval from baseline (ΔΔQTcF) in both the studies, being 3.29 (0.35, 6.23) ms at 40 mg and 5.27 (2.35, 8.20) ms at 120 mg dose in the TQT study, and 13.3 (8.1, 18.5) ms at 200 mg and 9.9 (4.7, 15.1) ms at 240 mg doses in the SAD study (all doses expressed as pitolisant hydrochloride). Corresponding pharmacokinetics provided C_max_ values of 53 ± 27, 164 ± 63 and 282 ± 47 ng·mL^−1^ at 40, 120 and 240 mg respectively. Thus, pitolisant, at the highest clinically relevant exposure of 35 and 72 ng·mL^−1^ (0.12 and 0.24 μM) at 20 and 40 mg daily dosing, respectively, lacked an effect of regulatory concern on QTc interval. Notwithstanding, it is acknowledged that to fully support CiPA, one would need additional data in the clinic, particularly in wider patient populations. Our findings are in agreement with the findings by Vargas *et al*. (2015) who reported that 91% of the drugs that prolong the QT interval in humans also did so in animals (guinea pigs, rabbits, dogs and non‐human primates), and 88% of the drugs that did not prolong the QT interval in humans also did not prolong the interval in animals.

In conclusion, our findings lend support to the CiPA initiative and suggest that the additional effects at calcium and sodium channels may enable a distinction to be made between QT interval prolongation that may be pro‐arrhythmic or antiarrhythmic. However, demonstrating inhibitions of the calcium and/or late I_Na_ current appears to be only one *in vitro* component for the demonstration of the lack of pro‐arrhythmic potential of a drug that may prolong the QT interval. The present study suggests that sponsors should consider investigating drug effects on EADs and the use of pro‐arrhythmia models (such as the Carlsson rabbit model) when the results from CiPA studies are ambiguous.

## Author contributions

X.L., I.B.B., G.R.M., P.R., L.L. and J.F.F. performed the research. X.L., I.B.B., P.R., L.L. and J.F.F. designed the research study. All authors contributed to analysis of data and drafting and revising of the manuscript. All authors have approved the final version of the article and are accountable for all aspects of the work.

## Conflict of interest

R.R.S. and P.M.B. are paid consultants to Bioprojet. P.M.B. is also a consultant to CARDIABASE, the centralized laboratory that analysed the ECGs from the two clinical studies and is currently also involved in drug profile selection for CiPA validation, acting as a clinical expert. G.R.M. is an academic scientist with no commercial affiliations but has provided advice to the FDA on *in silico* action potential modelling as part of the CiPA initiative. X.L., I.B.B., P.R. and L.L. are paid employees of Bioprojet‐Biotech. J.F.F. was a paid employee of Bioprojet‐Biotech until August 2004. J.M.L. and J.C.S. are shareholders of Bioprojet. The authors declare that they have no conflict of interest. The funding sources did not influence design and conduct of the study; collection, management, analysis and interpretation of the data and preparation, review or approval of the manuscript.

## Declaration of transparency and scientific rigour

This Declaration acknowledges that this paper adheres to the principles for transparent reporting and scientific rigour of preclinical research recommended by funding agencies, publishers and other organisations engaged with supporting research.

## Supporting information


**Table S1** Composition of intracellular solutions used to fill pipette to record currents through Na_V_1.5, K_V_4.3, K_V_7.1/mink, K_ir_2.1, K_V_1.5, Ca_V_1.2 and Ca_V_3.2 channels.
**Table S2** Stimulation conditions used to record currents through Na_V_1.5, K_V_4.3, K_V_7.1/mink, K_ir_2.1, K_V_1.5, Ca_V_1.2 and Ca_V_3.2 channels.
**Figure S1** Simulated effects of pitolisant on 1 Hz rabbit cardiomyocyte action potential parameters using the Shannon *et al.* (2004) model.
