# Supplementary material for: Nonclinical cardiovascular safety of pitolisant: comparing International Conference on Harmonization S7B and Comprehensive in vitro Pro‐arrhythmia Assay initiative studies
Source: Br J Pharmacol. 2017 Oct 19;174(23):4449–63. doi: 10.1111/bph.14047 (PMC5715595; doi:10.1111/bph.14047)
Supplement: Supplementary file 1 — Table S1 Composition of intracellular solutions used to fill pipette to record currents through NaV1.5, KV4.3, KV7.1/mink, Kir2.1, KV1.5, CaV1.2 and CaV3.2 channels. Table S2 Stimulation conditions used to record currents through NaV1.5, KV4.3, KV7.1/mink, Kir2.1, KV1.5, CaV1.2 and CaV3.2 channels. Figure S1 Simulated effects of pitolisant on 1 Hz rabbit cardiomyocyte action potential parameters using the Shannon et al. (2004) model. [file BPH-174-4449-s001.pdf]

Supporting Information

Nonclinical Cardiovascular Safety of Pitolisant: *Comparing ICH S7B and CiPA Initiative Studies*

Ligneau X *et al.*

Manuscript: 2017-BJP-0578-RP.R4

==

In vitro studies on other ion channels

The whole cell patch clamp technique was used to investigate the effect of pitolisant on currents through Nav1.5, Kv4.3, Kv7.1/mink, Kir2.1, Kv1.5, Cav1.2 and Cav3.2 channels.

HEK-293 or CHO cells were stably transfected with the different ion channels and maintained in 35-mm dish culture in the conditions mentioned in standard cell culture conditions. Experiments were performed with a cell density enabling isolated cells for patch clamping. Cells were continuously superfused with extracellular medium (1-2 mL·minute^{-1}) at room temperature. Compositions of the extracellular medium was (in mM): NaCl 137, KCl 4, $CaCl_2$ 1.8, $MgCl_2$ 1, D-glucose 10, HEPES 10 and the final pH adjusted to 7.4±0.02 with NaOH for recording Nav1.5, Kv4.3, Kv7.1/mink, Kir2.1, Kv1.5 and Cav3.2 currents. It was (in mM): NaCl 110, KCl 4, $BaCl_2$ 20, $MgCl_2$ 1, D-glucose 20, HEPES 10 and the final pH adjusted to 7.4 ± 0.02 with NaOH for recording the Cav1.2 current. Compositions of the intracellular solution to fill the pipette are provided in the Table S1.

Table S1:

Composition of intracellular solutions used to fill pipette to record currents through Nav1.5, Kv4.3, Kv7.1/mink, Kir2.1, Kv1.5, Cav1.2 and Cav3.2 channels

Ion channel	Solution to fill the pipette (mM)				
	Nav1.5	Kv4.3, Kir2.1, Kv1.5	Kv7.1/mink	Cav1.2	Cav3.2
KCl		130			110
K-aspartate			135		
CsF	135				
CsCl				80	
TEA-Cl				20	20
NaCl	10				
$MgCl_2$		1		4	1
EGTA	5	5	5	10	5
Na_2ATP			2		
MgATP		5		5	5
Na_2-phosphocreatine			14		
Phosphocreatine-Tris				20	
Creatine-phospho kinase			50 $U \cdot mL^{-1}$		
PMA			0.01		
HEPES	10	10	10	10	10
pH*	7.3	7.2	7.2	7.2	7.2

* pH adjusted with KOH.

After formation of a Gigaohm seal between the patch electrodes and individual cells (pipette resistance range: 2.0 MΩ - 7.0 MΩ; seal resistance range: > 1 GΩ) the cell membrane across the pipette tip was ruptured to assure electrical access to the cell interior (whole-cell patch-configuration). In case the quality of the seal was poor, the process of seal formation was repeated with a different cell and a new pipette. As soon as a stable seal was established outward tail currents were measured upon pulses described in the Table S2. If current density was judged to be too low for measurement, another cell was recorded. Once control recordings had been accomplished, cells were continuously perfused with a bath solution containing pitolisant and the voltage stimulation protocol indicated in the next table continuously run until the steady-state level of block was reached.

Table S2:

Stimulation conditions used to record currents through Nav1.5, Kv4.3, Kv7.1/mink, Kir2.1, Kv1.5, Cav1.2 and Cav3.2 channels

Ion channel	Holding potential	Pulse / duration	Frequency	Reference compound
$Na_V1.5$	-120 / -92 / 0 mV (resting / fast / slow inactivated	0 mV / 10 ms	0.05 Hz (between sweeps at one concentration) 0.0083 Hz between concentrations	Lidocaine (10 mM)
$K_V4.3$	-80 mV	+40 mV / 500 ms	0.1 Hz	Dapoxetine (30 µM)
$K_V7.1$/mink	-80 mV	+40 mV / 5000 ms	0.05 Hz	Mefloquine (10 µM)
Kir2.1	-60 mV	-120 +60 mV (ramp) / 1.0 s	0.1 Hz	ML 133 (10 µM)
$K_V1.5$	-80 mV	+40 mV / 5000 ms	0.05 Hz	Terfenadine (10 µM)
$Ca_V1.2$	-80 mV	+10 mV /300 ms	0.1 Hz	Nifedipine (1 µM)
$Ca_V3.2$	-80 mV and -120 mV (2 s before depolarisation)	-25 mV /250 ms	0.1 Hz	Mibefradil (1 µM)

Figure S1:

Simulated effects of pitolisant on 1 Hz rabbit cardiomyocyte action potential parameters using the Shannon *et al. (2004)* model.

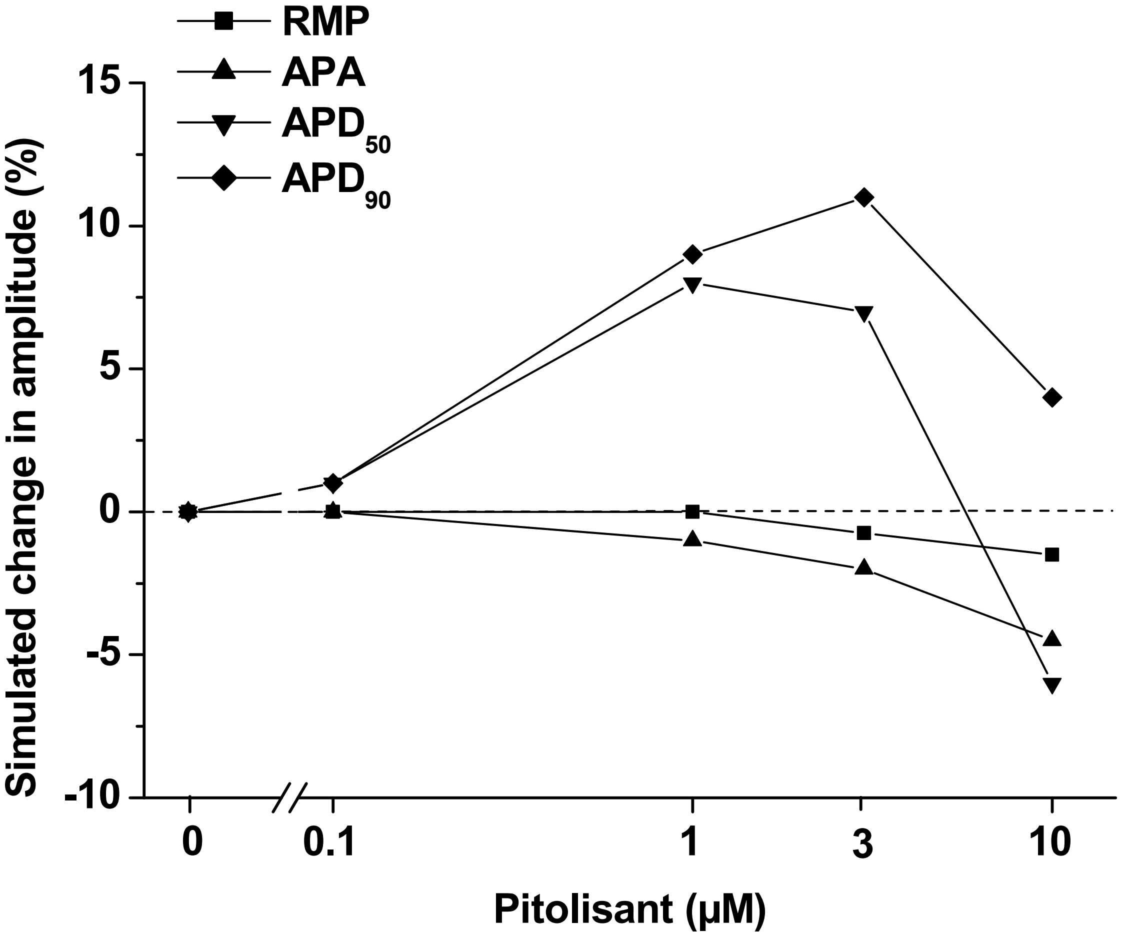

Parameters presented are resting membrane potential (RMP), action potential amplitude (APA), action potential durations measured at 50% and 90% of repolarisation (APD_{50} and APD_{90}).
